# Cytoplasmic ATR Activation Promotes Vaccinia Virus Genome Replication

**DOI:** 10.1016/j.celrep.2017.04.025

**Published:** 2017-05-02

**Authors:** Antonio Postigo, Amy E. Ramsden, Michael Howell, Michael Way

**Affiliations:** 1 Cellular Signalling and Cytoskeletal Function Laboratory, The Francis Crick Institute, 1 Midland Road, NW1 1AT London, UK; 2 High Throughput Screening Facility, The Francis Crick Institute, 1 Midland Road, NW1 1AT London, UK

**Keywords:** vaccinia virus, RPA, ATR, Chk1, DNA replication, PCNA, viral replisome

## Abstract

In contrast to most DNA viruses, poxviruses replicate their genomes in the cytoplasm without host involvement. We find that vaccinia virus induces cytoplasmic activation of ATR early during infection, before genome uncoating, which is unexpected because ATR plays a fundamental nuclear role in maintaining host genome integrity. ATR, RPA, INTS7, and Chk1 are recruited to cytoplasmic DNA viral factories, suggesting canonical ATR pathway activation. Consistent with this, pharmacological and RNAi-mediated inhibition of canonical ATR signaling suppresses genome replication. RPA and the sliding clamp PCNA interact with the viral polymerase E9 and are required for DNA replication. Moreover, the ATR activator TOPBP1 promotes genome replication and associates with the viral replisome component H5. Our study suggests that, in contrast to long-held beliefs, vaccinia recruits conserved components of the eukaryote DNA replication and repair machinery to amplify its genome in the host cytoplasm.

## Highlights

• Vaccinia activates cytoplasmic ATR early during infection and before genome uncoating
• Canonical ATR pathway activation promotes viral genome replication
• RPA is recruited to the viral genome
• PCNA, RPA, and TOPBP1 associate with the viral polymerase to promote DNA replication

## Graphical Abstract

**Figure:**
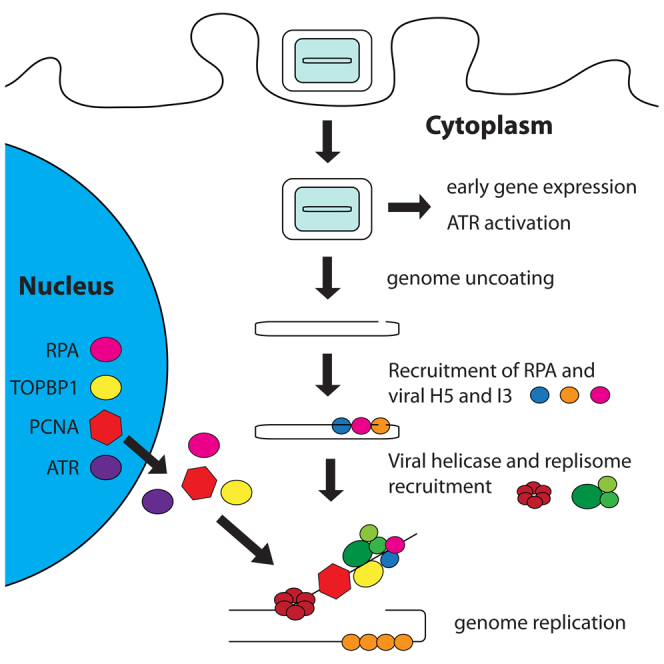

## Teaser

Postigo et al. suggest that, in contrast to long-held beliefs, vaccinia recruits conserved components of the eukaryote DNA replication and repair machinery to amplify its genome in the host cytoplasm.

## Introduction

Poxviruses such as vaccinia virus are complex enveloped viruses with large, linear, double-stranded DNA genomes that are covalently linked by hairpins at their inverted terminal repeats (Moss, 2013). In contrast to most other large DNA viruses, their genomes are replicated in cytoplasmic viral factories located near the nucleus through a mechanism that is still not understood (Boyle et al., 2015, Moss, 2013, Senkevich et al., 2015). The commonly held model is that replication proceeds via single-strand displacement (rolling circle replication), initiated from the genome termini (Du and Traktman, 1996, Pogo et al., 1984). The origin and identity of the nicked DNA sequence required to initiate replication remain unidentified. However, a study suggests replication is initiated at a single site near one of the terminal repeats and proceeds via semi-discontinuous rather than rolling circle replication (Senkevich et al., 2015). Regardless of the mechanism, it is thought that cytoplasmic vaccinia genome replication is largely independent of the host DNA replication machinery (Moss, 2013, Prescott et al., 1971). The virus encodes numerous DNA-modifying enzymes, including the essential core viral replisome components E9 (DNA polymerase), D5 (primase-helicase), D4 (uracil-DNA glycosylase), and A20 (accessory protein), all of which could allow for autonomous genome replication (Moss, 2013).

It has become apparent that the DNA damage response (DDR) plays a role not only in virus replication but also in viral detection (Ryan et al., 2016, Trigg and Ferguson, 2015, Turnell and Grand, 2012). For example, it is critical in promoting the replication of HIV, papilloma, herpes, and polyomaviruses in the nucleus (Luftig, 2014, Turnell and Grand, 2012). However, the DNA damage response can also suppress infection and consequently is often targeted by viral proteins (Turnell and Grand, 2012, Weitzman and Weitzman, 2014). The DNA damage response describes signaling pathways initiated by the related kinases ATR, ATM, and DNA-PK, which allow repair of single-stranded DNA (ssDNA; ATR) and double-stranded DNA (ATM and DNA-PK) breaks and initiation of cell death if the cell is beyond redemption (Awasthi et al., 2015, Ciccia and Elledge, 2010, Shiotani and Zou, 2009). DNA damage response proteins are predominantly located in the nucleus, where most DNA viruses replicate, so it is surprising that DNA-PK acts as a DNA sensor during cytoplasmic replication of vaccinia (Ferguson et al., 2012). Given this role for DNA-PK, we investigated whether ATM and ATR might also play a role during vaccinia replication.

## Results

### Vaccinia Activates ATM and ATR in the Cytoplasm

**Figure 1:**
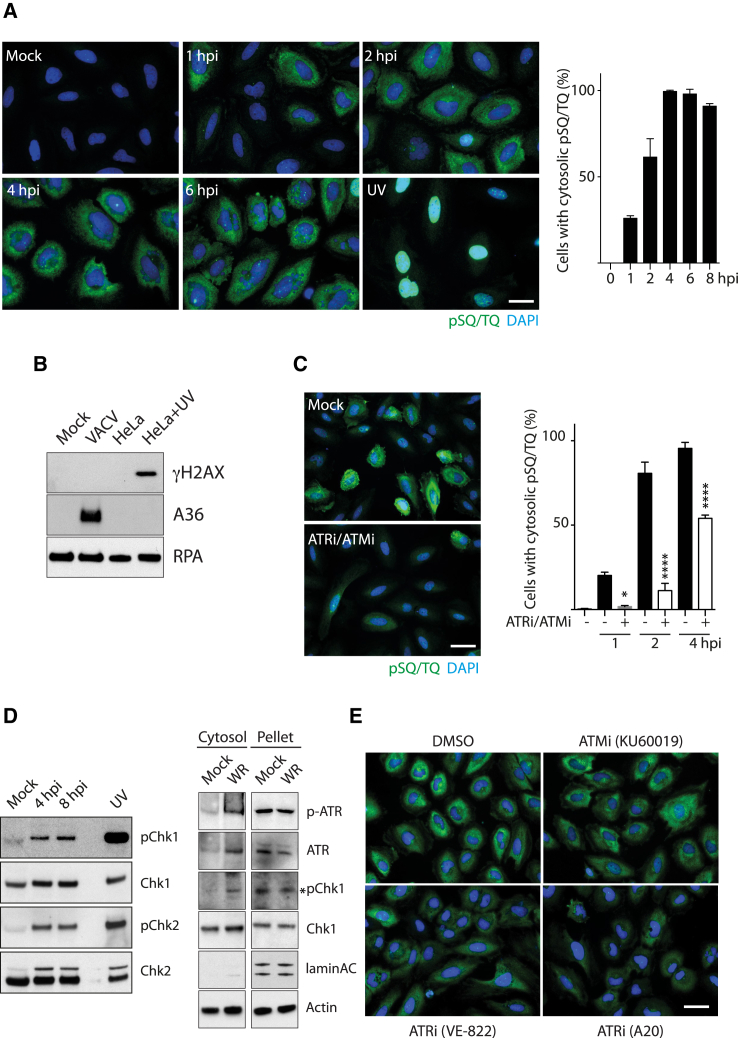
Vaccinia Induces a Cytoplasmic ATR/ATM-Dependent DNA Damage Response (A) Analysis of pSQ/TQ immunoreactivity (green) reveals phosphorylation of ATR/ATM substrates in response to infection with the Western Reserve (WR) strain of vaccinia virus at the indicated time post-infection or UV irradiation. The graph shows the quantitation of cells with cytoplasmic pSQ/TQ immunoreactivity. (B) Immunoblot analysis shows H2AX is phosphorylated in HeLa cells in response to UV irradiation, but not vaccinia infection. (C) Combined inhibition of ATM and ATR blocks vaccinia-induced cytoplasmic pSQ/TQ immunoreactivity. (D) Immunoblot analysis demonstrates that vaccinia induces phosphorylation of Chk1 and Chk2. Cell fractionation demonstrates that ATR and Chk1 are phosphorylated in the cytoplasm. (E) Analysis of pSQ/TQ immunoreactivity in cells infected for 4 hr and treated with the indicated inhibitors. All error bars represent SEM from three independent experiments in which a minimum of 200 cells were counted, with ^∗^p < 0.05 and ^∗∗∗∗^p < 0.0001. Scale bars, 20 μm.

Activation of ATR and ATM results in the phosphorylation of hundreds of substrates that contain a conserved SQ/TQ motif, including the checkpoint kinases Chk1 and Chk2 (Matsuoka et al., 2007, Traven and Heierhorst, 2005). Taking advantage of this, we examined the phosphorylation status of SQ/TQ motif-containing proteins in HeLa cells to investigate whether the ATR/ATM arms of the DNA damage response are activated by infection with the Western Reserve (WR) strain of vaccinia virus. As expected, UV irradiation induces an increase in phosphorylation of SQ/TQ motif-containing proteins, but only in the nucleus (Figure 1A). In contrast, from 1 hr post-infection onward, increasing numbers of infected cells display a prominent cytoplasmic pSQ/TQ signal (Figure 1A). This increase is not due to nuclear DNA damage because this increase is only observed in the cytoplasm (Figure S1). Furthermore, phosphorylated H2AX is observed only in UV-treated cells, not in infected cells (Figure 1B). The pSQ/TQ signal is specific for ATR/ATM activation, because it was abrogated by the combined treatment with the ATM inhibitor (ATMi) and ATR inhibitor (ATRi) (Figure 1C). Vaccinia-induced phosphorylation of Chk1 and Chk2 further established that infection activates the ATR and ATM pathways (Figure 1D). Moreover, phosphorylation of Chk1 and ATR largely occurs in the cytoplasm of infected cells (Figure 1D). Finally, treatment with two independent inhibitors demonstrated that the bulk of the pSQ/TQ signal is accounted for by ATR activity (Figure 1E).

### ATR Activation Is Independent of Viral Genome Uncoating

**Figure 2:**
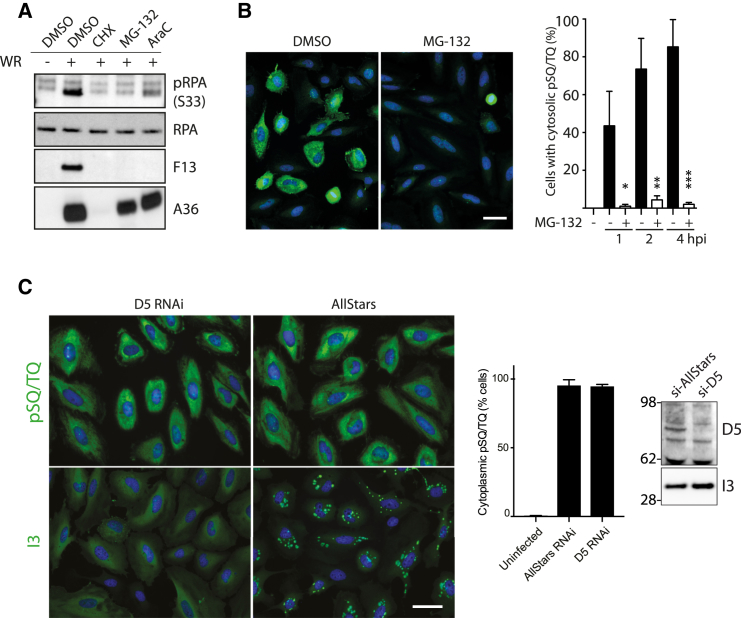
ATR Activation Is Independent of Viral Genome Uncoating (A) Immunoblot analysis reveals that cycloheximide (CHX) and the proteasome inhibitor MG-132, but not AraC, inhibit both RPA Ser33 phosphorylation and late viral protein expression (F13). (B) Quantitative immunofluorescence analysis reveals that MG-132 inhibits vaccinia-induced cytoplasmic pSQ/TQ immunoreactivity (green). (C) Quantitative immunofluorescence analysis reveals that loss of D5 does not inhibit vaccinia-induced cytoplasmic pSQ/TQ immunoreactivity or expression of I3. All error bars represent SEM from three independent experiments in which a minimum of 200 cells were counted, with ^∗^p < 0.05, ^∗∗^p < 0.005, and ^∗∗∗^p < 0.001. Scale bars, 20 μm.

An infection-induced DNA damage response can be stimulated by viral genomes that are detected as damaged DNA or by replicating genomes (Luftig, 2014, Weitzman and Weitzman, 2014). Alternatively, the DNA damage response can be directly activated by viral proteins such as L-Tag and E1 from Simian virus 40 (SV40) and human papillomavirus (HPV), respectively (Luftig, 2014, Weitzman and Weitzman, 2014). To begin to investigate how vaccinia induces the ATR pathway, we examined the level of serine 33 (Ser33) phosphorylation in RPA2 in infected cells treated with inhibitors blocking early gene expression (cycloheximide [CHX]), genome uncoating (MG-132), or genome replication (AraC). Ser33 in RPA2 is a direct ATR substrate (Vassin et al., 2004). AraC treatment reduced, but did not fully block, RPA2 phosphorylation (Figure 2A). In contrast, CHX and MG-132 inhibited Ser33 phosphorylation (Figure 2A), while MG-132 also blocked virus-induced phosphorylation of SQ/TQ motifs in the cytoplasm (Figure 2B). This suggests that a step post-genome release into the cytoplasm is responsible for activating ATR signaling (Figure 2A). However, MG-132 was reported to also affect an earlier stage in the life cycle, namely, disassembly of lateral bodies (Schmidt et al., 2013). Therefore, we investigated the requirement for uncoating in pSQ/TQ activation by small interfering RNA (siRNA)-mediated knockdown of D5, the viral primase-helicase required for uncoating (Kilcher et al., 2014). In contrast to MG-132 treatment, knockdown of D5 did not inhibit the appearance of cytoplasmic phosphorylated SQ/TQ motifs (Figure 2C). Loss of D5 also had no impact on the expression of the viral DNA binding protein I3, which in the absence of virus replication, remained diffuse in the cytoplasm (Figure 2C). The most straightforward explanation for our observations is that activation of ATR signaling occurs before and independently of viral genome uncoating and replication.

### ATR and Chk1 Are Required for Vaccinia Genome Replication

**Figure 3:**
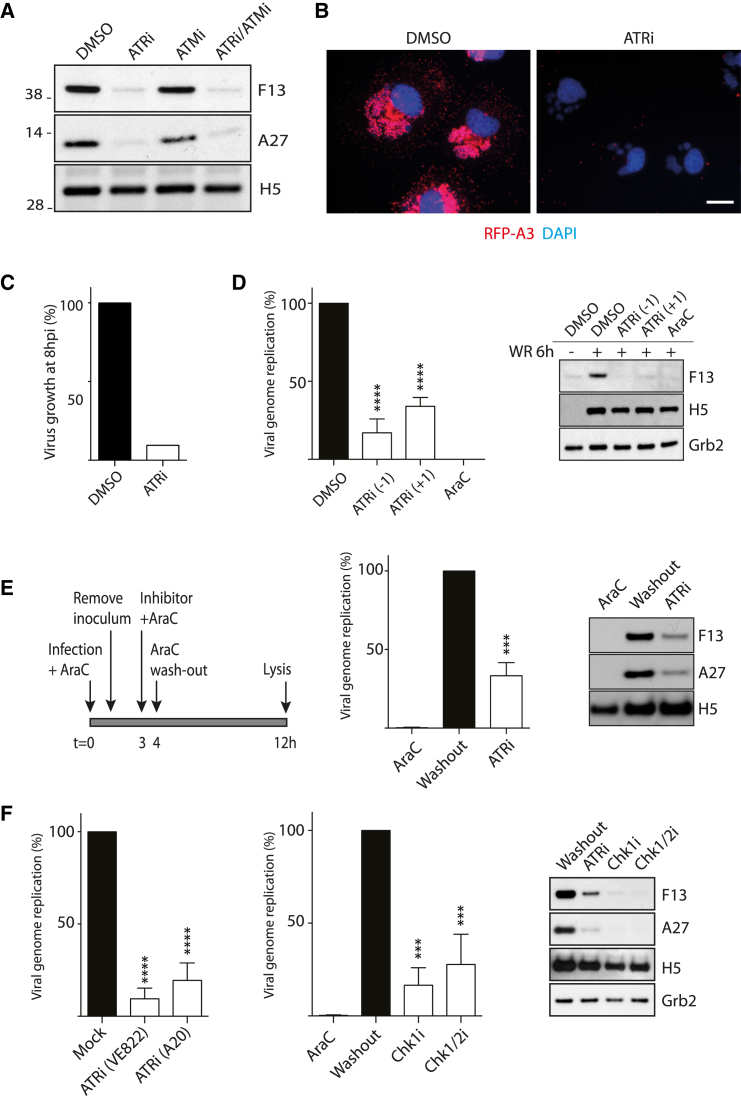
ATR and Chk1 Are Required for Vaccinia Genome Replication (A) Immunoblot analysis demonstrates that ATR (ATRi VE-821), but not ATM (ATMi KU55633), inhibits late (F13 and A27), but not early (H5), viral protein expression 8 hr post-infection. (B) Immunofluorescence analysis of cells infected for 8 hr reveals that ATRi inhibits expression of RFP-A3, a late viral core protein. Scale bar, 10 μm. (C) ATRi inhibits viral production 8 hpi. (D) Quantification of viral genome copy number in cells infected for 6 hr in the presence of AraC or ATRi 1 hr pre- (−1) or post- (+1) infection. Both AraC and ATRi inhibit expression of F13 (late), but not H5 (early). (E) Removal of AraC 4 hr post-infection in the presence, but not absence, of ATRi reduces genome replication and late (F13, A27), but not early (H5), viral protein expression. (F) Removal of AraC 4 hr post-infection in the presence of the indicated inhibitors reduces genome replication and late gene expression. All error bars represent SEM from three independent experiments, with ^∗∗∗^p < 0.001 and ^∗∗∗∗^p < 0.0001.

To investigate whether ATR/ATM activation represents a response to vaccinia infection or is required for viral replication, we examined the impact of chemical inhibitors of the two kinases (ATMi and ATRi) on infected cells. We found that inhibition of ATR, but not ATM, suppressed late viral protein expression, which is dependent on vaccinia DNA replication (Figure 3A). Immunofluorescence analysis confirmed that treatment of infected cells with ATRi inhibited late viral protein expression, as well as virus factory formation (Figure 3B). Consistent with this, inhibition of ATR decreases viral production 10-fold 8 hr post-infection (Figure 3C). Furthermore, direct measurement of genome copy number by real-time PCR confirms that inhibition of ATR 1 hr before or after infection significantly decreases viral DNA replication (Figure 3D). This inhibition was not as complete as the replication inhibitor AraC, although both drugs block late viral protein expression (Figure 3D). This suggests that ATR is required for DNA replication but does not exclude the possibility that it acts before DNA synthesis. To determine whether ATR is necessary for viral DNA replication, we infected cells for 4 hr in the presence of AraC before washing out the drug in the absence or presence of ATRi (Figure 3E). Following AraC washout in the absence of ATRi, both early (H5) and late (F13 and A27) protein expression were detected 12 hr post-infection (Figure 3E). There was also a concomitant increase in viral genome replication, consistent with the recommencement of a stalled replication cycle (Figure 3E). In contrast, after AraC removal in the presence of ATRi, late gene expression and genome replication remained suppressed (Figure 3E). Early protein expression (H5) appeared unaffected. Similar results were obtained with two additional ATRis, as well as inhibitors of Chk1 and Chk1/2 (Figure 3F). Altogether, our observations demonstrate that the activation of Chk1 downstream of ATR facilitates cytoplasmic viral genome replication.

### ATR Signaling Mediates Cytoplasmic Genome Replication

**Figure 4:**
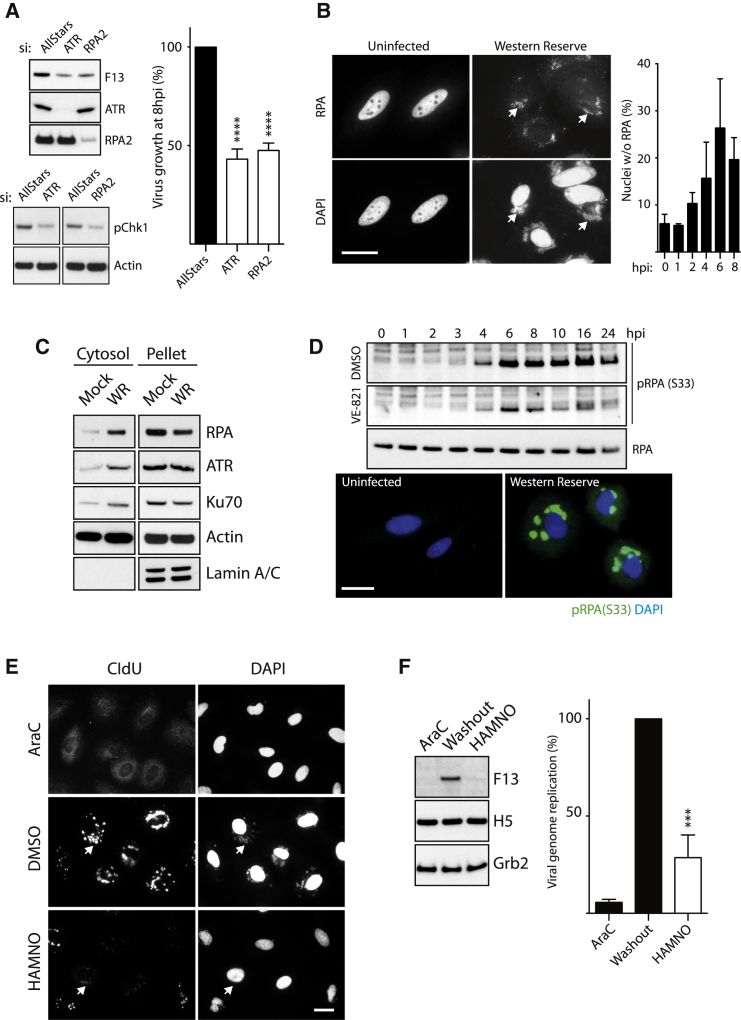
RPA2 Recruitment of ATR Mediates Cytoplasmic Genome Replication (A) Analysis of F13 expression and virus growth in ATR- and RPA2-depleted HeLa cells 8 hr post-infection. (B) Immunofluorescence images reveal RPA is recruited to viral factories 8 hr post-infection (arrowheads). Quantitation of subcellular localization of RPA shows nuclear depletion of RPA2 during infection. (C) Immunoblot analysis of fractionated cells reveals that vaccinia increases the level of RPA2, ATR, and Ku70 in the cytoplasm 8 hr after infection. (D) Immunoblot analysis of whole-cell extracts reveals vaccinia induces phosphorylation of Ser33 of RPA2 from 4 hr post-infection. Immunofluorescence images showing pRPA2 (Ser33) co-localizes with viral factories 4 hr post-infection (green). (E) Immunofluorescence analysis of CldU incorporation after AraC washout reveals HAMNO treatment suppresses DNA replication and viral factory formation (arrowheads). (F) HAMNO inhibits viral genome replication, as well as late viral gene expression 12 hpi. All error bars represent SEM from three independent experiments, with ^∗∗∗^p < 0.001 and ^∗∗∗∗^p < 0.0001. Scale bars, 20 μm.

ATR is activated by DNA lesions that generate ssDNA and by stalled replication forks in a process that is incompletely understood (Cimprich and Cortez, 2008, Maréchal and Zou, 2013). The recruitment of ATR to these sites is dependent on the trimeric ssDNA-binding RPA1/2/3 complex bound to ssDNA (Zou and Elledge, 2003). Consistent with a role in recruiting ATR, we found that RNAi-mediated depletion of RPA2 results in a similar decrease in late viral gene expression, virus growth, and Chk1 phosphorylation as loss of ATR (Figure 4A). The ATR knockdown result also confirms that the two independent ATRis did not act by inhibiting a viral protein. In non-infected cells, RPA2 is located in the nucleus (Figure 4B). After infection, cells show a progressive decrease in nuclear localization of RPA and a concomitant increased association of the protein with cytoplasmic DNA factories (Figure 4B). The inhibition of CRM1-dependent nuclear export had no impact on redistribution of RPA to the cytoplasm, virus growth, or genome replication (Figure S2A). Biochemical fractionation also established that RPA and ATR, as well as Ku70, part of the DNA-PK vaccinia DNA sensor complex (Ferguson et al., 2012), become significantly enriched in the cytoplasm during infection (Figure 4C). Like RPA, ATR, Chk1, and Ku70 associate with cytoplasmic viral DNA factories (Figure S2B).

Hyperphosphorylation of RPA2 is required for ATR recruitment to DNA breaks and stalled replication forks (Maréchal and Zou, 2015). Consistent with a role for activated ATR during infection, we found that Ser33 in RPA2, an ATR substrate, becomes phosphorylated in an ATR-dependent manner from 4 hr post-infection at the time of genome replication (Figure 4D). ATR-dependent RPA phosphorylation was also associated with cytoplasmic DNA factories (Figure 4D). The inhibitor HAMNO disrupts the interaction of RPA with effectors such as ATR while leaving RPA-ssDNA interactions intact (Glanzer et al., 2014). In an AraC washout experiment, HAMNO dramatically decreases viral DNA replication based on the lack of viral factories with incorporated 5-chloro-2′-deoxyuridine (CldU) (Figure 4E). HAMNO also suppresses late gene expression and viral genome replication (Figure 4F). This confirms that RPA is required to recruit ATR for genome replication.

**Figure 5:**
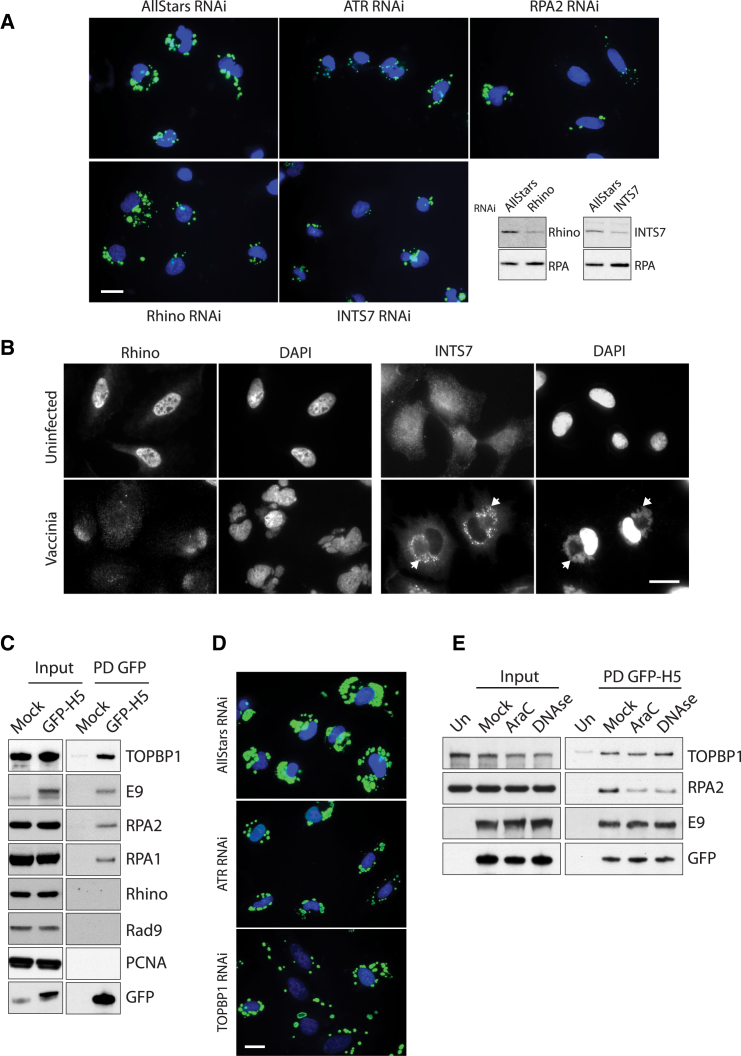
Viral Genomes Recruit Replisome-Associated RPA2 and Activate ATR (A) Immunofluorescence analysis of cells infected for 4 hr reveals a reduced incorporation of CldU (green) into developing viral factories in cell treated with siRNA against ATR, RPA2, Rhino, and INTS7. (B) Rhino is re-localized from the nucleus to the cytoplasm after infection, while INTS7 co-localizes with viral DNA factories 8 hr post-infection (arrowheads). (C) Immunoblot analysis of GFP-Trap pull-downs on cells infected with GFP-H5 virus for 5 hr. (D) Knockdown of ATR and TOPBP1 reduces incorporation of CldU (green) into developing viral factories. (E) Immunoblot analysis of GFP-Trap pull-downs on cells infected with GFP-H5 virus for 5 hr in the presence of AraC or after lysates were treated with DNase I. Scale bars, 20 μm.

Consistent with our observations, we found that knockdown of Rhino and INTS7, two proteins that are required for full ATR pathway activation (Cotta-Ramusino et al., 2011), also reduces viral DNA replication (Figure 5A). Moreover, upon infection, INTS7 co-localizes with viral DNA factories, while Rhino re-distributes from the nucleus into the cytoplasm (Figure 5B). The role of Rhino is to stabilize the interaction between TOPBP1 and ATR to facilitate full activation of ATR (Cotta-Ramusino et al., 2011, Lindsey-Boltz et al., 2015). Pull-downs on lysates from cells infected with a recombinant virus expressing GFP-H5 demonstrate that TOPBP1, E9, and RPA1/2 formed a complex with H5 (Figure 5C). This interaction is not mediated by the GFP tag, because it is not seen with GFP-tagged F12, a viral protein involved in virus assembly (Figure S3) (Dodding et al., 2009). In contrast, Rhino and Rad9, which are involved in recruiting and activating ATR, did not form part of this complex, nor did PCNA, a replication-associated protein (Figure 5C). Consistent with a role for TOPBP1 in ATR-mediated genome replication, we found that loss of TOPBP1 also impaired viral DNA replication (Figure 5D). Inhibition of replication by AraC reduced the association of H5 with RPA, but not with TOPBP1 or E9 (Figure 5E). Moreover, treatment of the pull-down with DNase I reduced the association of H5 with RPA, but not with E9 and TOPBP1 (Figure 5E). Our data suggest that RPA is recruited to a H5/E9/TOPBP1 complex via viral DNA.

### Viral Genomes Recruit RPA and PCNA as Part of the Viral Replisome

**Figure 6:**
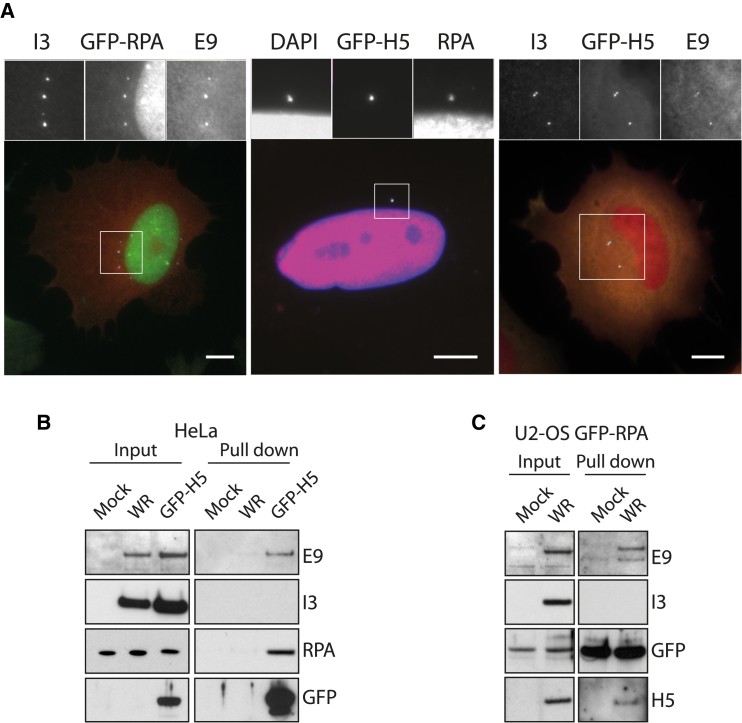
Viral Genomes Recruit Replisome-Associated RPA2 (A) Immunofluorescence analysis reveals that RPA co-localizes with H5, I3, and E9 on viral genomes in cells infected for 6 hr in the presence of AraC. Scale bars, 20 μm. (B) Immunoblot analysis of GFP-Trap pull-downs from cells infected for 5 hr with GFP-H5 virus reveals that RPA, but not I3, associates with the viral replisome components E9 and H5. (C) Immunoblot analysis of GFP-Trap pull-downs performed on lysates from U2-OS-GFP-RPA cells infected for 5 hr with WR reveals that RPA, but not I3, associates with the viral replisome.

Once released into the cytoplasm, the vaccinia genome is bound by the viral proteins H5 and I3 (Beaud and Beaud, 1997, Rochester and Traktman, 1998). I3 is a ssDNA-binding protein that contributes to genome replication (Greseth et al., 2012), while H5 associates with the E9/D4/A20 holoenzyme and DNA in vitro. H5 is required for genome replication, though its function is unknown (Boyle et al., 2015). We found that GFP-H5 co-localizes with the E9 DNA polymerase and I3 on replication-inhibited genomes in AraC-treated infected cells (Figure 6A). Moreover, RPA co-localizes on H5-positive viral genomes in the cytoplasm of AraC-treated cells (Figure 6A). We could also detect RPA, together with E9 and I3, on viral genomes in infected U2OS cells stably expressing GFP-RPA in the presence of AraC (Figure 6A). In pull-down experiments, RPA and E9 interact with GFP-H5, but not with I3 (Figure 6B). GFP-RPA also forms complexes with E9 and H5, but not I3 (Figure 6B). AraC-mediated replication inhibition diminishes ATR-dependent phosphorylation of RPA (Figure 2A) and the amount of RPA associating with the GFP-H5/E9/TOPBP1 complex (Figure 5E), consistent with a role for RPA in replisome activity. Altogether, this suggests that RPA, rather than I3, is the replicative ssDNA-binding protein associated with the vaccinia replisome.

**Figure 7:**
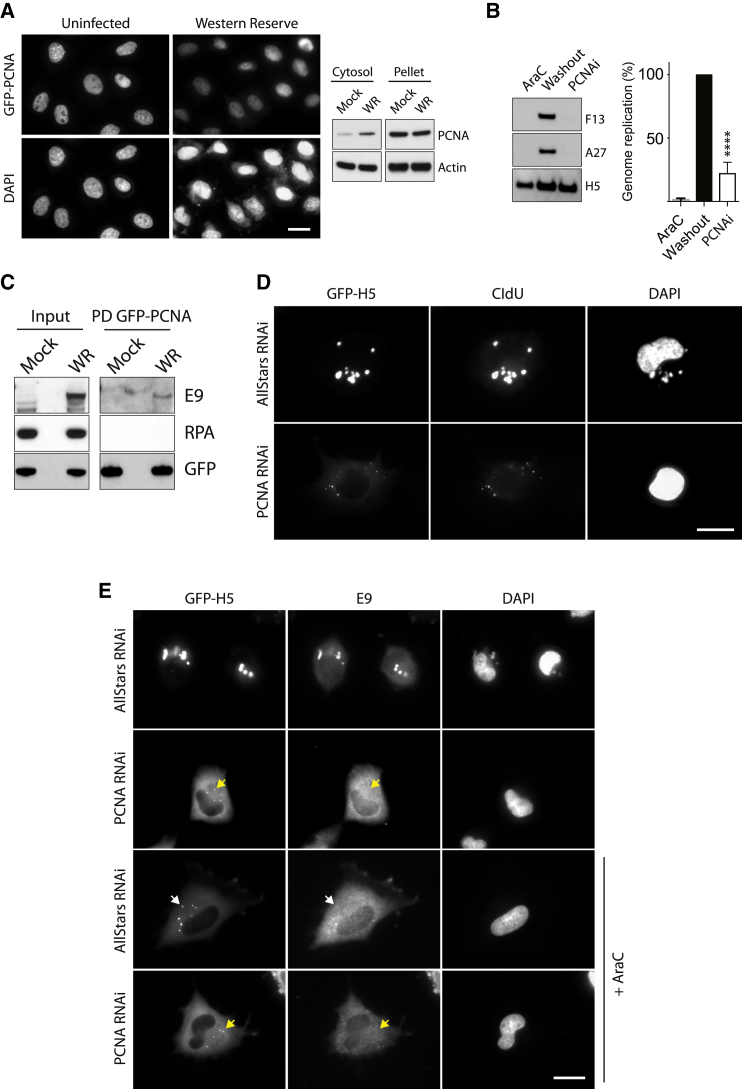
PCNA Is Required for Viral DNA Replication (A) Immunofluorescence analysis reveals that infection with WR decreases the level of GFP-PCNA in the nucleus. Immunoblot analysis shows a corresponding increase of PCNA in the cytoplasm. (B) Inhibition of PCNA (PCNAi) reduces genome replication and late viral protein expression (F13 and A27), but early viral protein expression (H5) after AraC washout appears unaffected. Error bars represent SEM from three independent experiments, with ^∗∗∗∗^p < 0.0001. (C) Immunoblot analysis of GFP-Trap pull-downs reveals GFP-PCNA associates with E9, but not RPA. (D) Immunofluorescence analysis of CldU incorporation reveals loss of PCNA impedes DNA replication and viral factory formation. (E) Immunofluorescence analysis of cells infected for 5 hr reveals that loss of PCNA abolishes co-localization of E9 with GFP-H5 on replicating and replication-inhibited (+AraC) viral factories (yellow arrowheads). Scale bars, 20 μm.

Vaccinia encodes its own DNA polymerase and primase-helicase; however, it lacks an obvious sliding clamp protein to strengthen the interaction between polymerase and DNA. Given this, and in light our observation that RPA and TOPBP1 are complexed with the viral replisome, we wondered whether the host sliding clamp PCNA is also recruited to replicating viral DNA. Consistent with this notion, we observed that PCNA accumulates in the cytoplasm of infected cells (Figure 7A). Chemical inhibition of PCNA blocked genome replication and late gene expression (Figure 7B). Next, we investigated the association of PCNA with the viral replisome. PCNA does not complex with GFP-H5 (Figure 5C). In contrast, GFP-PCNA associates with the E9 DNA polymerase but was unable to interact with RPA (Figure 7C). Moreover, RNAi-mediated ablation of PCNA dramatically reduced viral DNA replication (Figure 7D). It also abolished the co-localization of E9 with replicating genomes (Figure 7E). Replication-inhibited genomes in AraC-treated cells were also unable to recruit E9 in cells lacking PCNA (Figure 7E). These data suggest that PCNA enhances the processivity of the viral replisome by recruiting and stabilizing E9 on the viral genome after uncoating.

## Discussion

To undergo a productive infection in the cytoplasm, vaccinia virus must avoid host recognition of viral genomes by DNA sensor proteins and be able to replicate its DNA without access to the host nuclear DNA replication machinery. While it is firmly held that vaccinia encodes all proteins required to replicate its genome autonomously, how the virus avoids the deleterious impact of DNA sensing is not well understood. A study has shown that the DNA-PK complex is a sensor of vaccinia DNA leading to an anti-viral cytokine response (Ferguson et al., 2012). We have demonstrated that the related kinase ATR acts to promote virus replication as part of an infection-induced cytoplasmic ATR/ATM-dependent response. This cytoplasmic response is induced before genome uncoating, not by the sensing of uncoated genomes and/or their replication, because it is inhibited by RNAi-mediated loss of the viral primase-helicase D5 (Figure 2). Our observation with vaccinia contrasts the situation with viruses such as Minute Virus of Mice (MVM) parvovirus, in which a DNA damage response is only induced after replication commences (Adeyemi et al., 2010).

It remains to be understood how ATR activity promotes vaccinia genome replication. ATR could act directly on viral replisome or its associated proteins. However, the requirement of its canonical pathway via Chk1 and the localization, as well as the effect of knockdown of ATR pathway components (Chk1, INTS7, Rhino, and TOPBP1), parallel its role in eukaryotes, in which ATR function overcomes stalled replication fork progression (Awasthi et al., 2015, Ciccia and Elledge, 2010, Shiotani and Zou, 2009). This form of replication stress can be due to physical barriers such as telomeric or repetitive structures. This dislodges the tight connection between helicase and polymerase activity, resulting in long tracts of ssDNA that become coated by RPA, which in turn recruit ATR (Mazouzi et al., 2014). The inverted terminal repeats at the ends of the vaccinia genome contain large numbers of 70- and 54-base pair tandem repeats, which are A/T rich, so it is possible that ATR acts to facilitate replication through these sequences (Moss, 2013). These regions also contain several unpaired bases, which may also partly account for the early recruitment of the ssDNA-binding protein RPA that, together with ATR, is required for genome replication.

Our results demonstrate that RPA, but not I3, the viral ssDNA-binding protein, forms a complex with H5 and E9, core components of the viral replisome. The function of I3 in viral genome replication remains enigmatic (Greseth et al., 2012). The I3 knockdown leads to a 3- to 7-fold reduction in DNA replication; nevertheless, these genomes are correctly processed (Greseth et al., 2012). I3 is recruited to unknown ssDNA sequences that must be present after genomes uncoat. It is possible that I3 interacts with H5 and E9 independently of RPA, although such a separate complex has not been reported. The simultaneous requirement for both a host and a viral ssDNA binding protein for viral replisome activity would be unprecedented. We therefore suggest that RPA, and not I3, as previously proposed (Rochester and Traktman, 1998), is the replicative ssDNA-binding protein associated with the vaccinia replisome. Vaccinia replicates origin independently (De Silva and Moss, 2005), so the site or sites to which RPA initially binds in the genome are not immediately obvious. However, an attractive possibility is that RPA, together with an H5- and E9-containing replisome complex, pre-assembles at nicked DNA that is present or generated after uncoating (DeMasi et al., 2001, Senkevich et al., 2015). Moreover, our observations demonstrating that RPA, together with other host proteins involved in DNA replication and repair, is recruited out of the nucleus and associates with viral factories suggests it plays an important role in both the initiation and the propagation of viral DNA replication. The essential role of H5 in genome replication, though poorly understood, has been attributed to a scaffolding function. Our finding that H5 interacts with E9 and TOPBP1 supports this notion. However, to what extent H5 might mimic the activity of a host protein or proteins remains to be established.

Vaccinia does not encode a functional sliding clamp protein to strengthen the interaction between viral DNA and its polymerase (Beaud and Beaud, 1997). Our data offer an explanation for this observation, because vaccinia clearly requires the host sliding clamp protein PCNA to enhance its DNA replication. Infection results in the accumulation of PCNA in the cytoplasm. Nevertheless, we were unable to detect the protein on replicating DNA viral factories. This suggests that although GFP-tagged PCNA can associate with E9, it may not be fully functional to replicate DNA or that the level of its recruitment is below our detection limit. In-depth analysis of vaccinia replisome-associated components with more recently developed techniques such as isolation of proteins on nascent DNA (iPOND) (Sirbu et al., 2011) may confirm that PCNA is associated with replicating viral DNA and uncover additional replication-associated host proteins.

Previous studies have shown a number of DNA binding proteins, including DNA-PK, BAF, and DNA ligase I, as well as several transcriptional and translational regulators, are also present on viral factories (Ferguson et al., 2012, Katsafanas and Moss, 2007, Oh and Broyles, 2005, Paran et al., 2009, Wiebe and Traktman, 2007). An essential role for nuclear host proteins in viral DNA replication may explain why several nuclear pore complex proteins were shown to contribute to virus replication in siRNA screens (Mercer et al., 2012, Sivan et al., 2013). Moreover, loss of Nup62, a core component of the nuclear export pore, moderately affected DNA replication but severely inhibited viral morphogenesis (Sivan et al., 2013). Based on our observations and those of others, it is likely that the altered nucleo-cytoplasmic distribution and recruitment to virus factories of host proteins are indicative of their wider involvement in viral replication than previously appreciated. However, while advantageous, the recruitment of nuclear proteins may come at a price, when anti-viral factors normally restricted to the nucleus are able to detect and respond to cytoplasmically replicating virus. The opposite activities of the DNA-PK (anti-viral) and ATR (pro-viral) pathways illustrate this and may explain why vaccinia encodes C16, an inhibitor of DNA-PK activation (Peters et al., 2013).

In conclusion, our data dispel long-held beliefs concerning the lack of host involvement in vaccinia replication. Moreover, they suggest that processive vaccinia replisome activity shares more similarities with the eukaryote machinery than previously anticipated (Moss, 2013). The task ahead is to establish the mechanism by which vaccinia induces cytoplasmic ATR activation. Similarly, how ATR and PCNA activity outside the nucleus promotes replication needs further exploration.

## Experimental Procedures

### Cells and Viruses

HeLa and BS-C-1 cells were grown in minimum essential medium (MEM)/10% fetal calf serum (FCS). U2OS-GFP-RPA cells (a gift from Prof. Jiri Lukas) (Toledo et al., 2013) were cultured in DMEM/10% FCS and 400 μg/mL G418. Stable cells expressing GFP-tagged PCNA were generated by introducing GFP-PCNA (Addgene plasmid 21048) in HeLa Kyoto cells (a gift from Mark Petronczki, Boehringer Ingelheim). Recombinant vaccinia strains were generated in the strain WR: ΔF11L (Cordeiro et al., 2009), GFP-F12L (Dodding et al., 2009), and RFP-A3L (Weisswange et al., 2009) were previously reported. WR expressing GFP-H5 was constructed by insertion of GFP in front of the H5R open reading frame by homologous recombination using techniques similar to those previously reported (Weisswange et al., 2009). The fidelity of the insertion site was determined by DNA sequencing. All virus stocks were purified through a 36% sucrose cushion and quantified by plaque titration.

### Chemicals

Chemical inhibitors were obtained from the following suppliers and used at the indicated concentrations: KU55933 (ATMi) (Hickson et al., 2004) (118500, Millipore, 20 μM), VE-821 (ATRi) (Reaper et al., 2011) (A11605, Adooq Bioscience, 20 μM), SB218078 (Chk1i) (559402, Millipore, 5 μM), AZD7762 (Chk1/2i) (SML-0350, Sigma, 2 μM), MG-132 (Sigma, 25 μM), CHX (C7698, Sigma, 50 μM), AraC (C1768, Sigma, 50/5 μM), HAMNO (SML1234, Sigma, 20 μM), KU60019 (HY-12061, Insight Biotechnology, 10 μM), VE-822 (10 μM), AZ20 (Foote et al., 2013) (HY-15557, Insight Biotechnology, 15 μM), T2AA (SML0794, Sigma, 20 μM), KPT-251 (5005050001, Millipore, 20 μM), bortezomib (2 μM), carfilzomib (17554, Cambridge Bioscience, 2 μM), and CldU (C6891, Sigma, 5 μM).

### Viral Growth Assays

HeLa cells (2 × 10^5^) were plated on fibronectin-coated 6-well plates 24 hr before infection with vaccinia virus at an MOI of 4. At 1 hours post infection (hpi), the serum-free media were changed to serum-containing media. Samples were taken 8 hr post-infection by scraping the cells in the media. After three freeze-and-thaw cycles, the samples were sonicated in a water bath and titered on BS-C-1 cells. ATRi (VE-821) and KPT-251 were added from 1 hr before infection. For RNAi studies, HeLa cells were transfected with 50 nM All-Star control (SI03650318, QIAGEN), ATR siRNA (si-ATR), and RPA siRNA (si-RPA) using Hiperfect (QIAGEN) 72 hr before experimentation, as per the manufacturer’s instructions. The following combinations of siRNA were used: si-ATR, 5′-CCUCCGUGAUGUUGCUUGA-3′ and 5′-CCUCCGUGAUGUUGCUUGA-3′; si-Rhino, 5′-CCGAGGACAAGUAUGGAAUAA-3′ and 5′-ACCACUACUCAUUAAUCCUUA-3′; si-INTS7, 5′-CAGCACGGAUCUAAACCAGGA-3′ and 5′-CAGCGUCAUCUUUGGUUGAUA-3′; si-PCNA, 5′-UAUGGUAACAGCUUCCUCC-3′ and 5′-CGGUGACACUCAGUAUGUC-3′; si si-TOPBP1, 5′-AGACCUUAAUGUAUCAGUA-3′; and si-RPA2, 5′-CCUAGUUUCACAAUCUGUU-3′. Vaccinia D5 was knocked down by transfecting HeLa cells twice with 100 μM si-D5 5′-CGUAACACCUUGUGCAUUA-3′ (Kilcher et al., 2014) 48 and 24 hr before infection. Efficiency of knockdown was determined using I3 immunofluorescence and/or immunoblot analysis. All oligonucleotides were made by Sigma.

### Antibodies, Pull-Downs, and Immunoblot Analysis

Cells were lysed in sample buffer (125 mM Tris-HCl, 4% SDS, 20% glycerol, 10% β-mercaptoethanol), containing 10 mM NaF whenever phopho-blots were performed. Proteins were separated on 4%–12% gradient Bis-Tris NuPAGE gels (Life Technologies) and transferred to nitrocellulose membranes. Immunoblots were incubated with antibodies detecting ATR (Santa Cruz Biotechnology, SC-1887), RPA2 (Santa Cruz Biotechnology, SC-56770), RPA1 (New England Biolabs, 2267), β-actin (Sigma, A5316), RPA phospho-Ser33 (Bethyl Laboratories, A300-246A), Grb2 (BD Bioscience, 610112), Lamin A/C (Santa Cruz Biotechnology, SC-7269), Ku70 (Santa Cruz Biotechnology, SC-12729), TOPBP1 (Bethyl Laboratories, A300-111), Rhino (Novus Biologicals, NBP1-93694), INTS7 (GeneTex, GTX82516), PCNA (New England Biolabs, 13110), H2AX phospho-Ser139 (Abcam, ab2893), GFP (Santa Cruz Biotechnology, SC-8334), A27 (Rodriguez et al., 1985), E9 (Magee et al., 2009), F13 polyclonal (Rietdorf et al., 2001), I3 (Lin et al., 2008), D5 (Evans and Traktman, 1987), and H5 (Cudmore et al., 1996). For GFP-Trap pull-downs, HeLa cells were grown in 100 or 150 mm (GFP-PCNA pull-down) dishes, infected with vaccinia virus (MOI = 4) for 6 hr, and harvested in PBS. The cell pellet was then processed for pull-down analysis as per manufacturer’s instructions (ChromoTek).

### Immunofluorescence

For immunofluorescence studies, coverslips were pre-coated with fibronectin (Arakawa et al., 2007). Cells were fixed with 4% paraformaldehyde (PFA) for 10 min, permeabilized in 0.1% (v/v) Triton/PBS for 2 min, and blocked in blocking buffer (1 mM MES, 15 mM NaCl, 0.5 mM EGTA, 0.5 mM MgCl_2_, 0.5 mM glucose [pH 6.1]) containing 3% (v/v) FCS and 1% (w/v) BSA for 30 min. Cells were labeled with primary antibodies against Phospho-(Ser/Thr) ATM/ATR (New England Biolabs, 2851), RPA (Santa Cruz Biotechnology, SC-56770), Ku70 (Bethel Laboratories, IHC-00723), ATR (Bethel Laboratories, A300-138A), Chk1 (Bethyl Laboratories, IHC-00004), RPA phospho-Ser33 (Bethyl Laboratories, IHC-00421), INTS7 (GeneTex, GTX82516), E9 monoclonal (Magee et al., 2009), and I3 polyclonal (Welsch et al., 2003). Secondary antibodies were conjugated with Alexa 488, 555, or 647 (Life Technologies). Immunofluorescence images were acquired on a Photometrics Cool Snap HQ cooled charge-coupled device (CCD) camera attached to a Zeiss Axioplan2 microscope using 63 × 1.4 numerical aperture (NA) (Plan Achromat) or 100 × 1.3, 40 × 1.3, or 25 × 0.8 NA (Plan NeoFluar) objectives. The system was controlled with MetaMorph 6.3r7 software. Quantification of pSQ/TQ immunoreactivity was determined after infection with the ΔF11L virus, which does not undergo virus-induced cell contraction early during infection (Cordeiro et al., 2009, Morales et al., 2008). Bar graphs represent the average of three independent experiments in which a minimum of 200 cells derived from at least four random fields were counted. Graphs were compiled using Prism (GraphPad). All figures were generated using Adobe software.

### Genome Replication

For AraC washout assays, HeLa cells (1.5 × 10^5^) were split into 6-well plates. The next day, cells were infected (MOI = 4) in serum-free MEM in the presence of AraC (5 μM). The inoculum was replaced first 1 hr post-infection with AraC-containing media (MEM/10% FCS) and then 3 hr post-infection with inhibitor and AraC-containing media. The wells were washed three times with 1 mL inhibitor-containing media 4 hr post-infection and then incubated until 12 hr post-infection. Cells were subsequently lysed in sample buffer for immunoblot analysis or harvested by scraping in media for real-time PCR analysis.

Vaccinia genome copy number was determined as previously described (Dai et al., 2014). In short, cells were harvested and processed for DNA extraction using the DNeasy Mini kit (QIAGEN), as per the manufacturer’s instructions. Real-time PCR was performed on an Applied Biosystems 7500 Real-time PCR Instrument (Life Technologies) using primers and TaqMan probe specific for the I4L gene, as described in Liu et al. (2006). Viral copy numbers were calculated from obtained cycle threshold (CT) values that were plotted on a standard curve. A plasmid containing the cloned I4L gene was used to establish the standard curve. Quantitation consisted of averaging of a minimum of three independent experiments performed in triplicate, in which genome replication was expressed as a percentage of (AraC washout + DMSO).

### CldU Incorporation

In an AraC washout assay, cells were treated with the HAMNO inhibitor (20 μM) as described earlier. At 10 hr post-infection, the medium was changed to additionally contain 5 μM CldU. Two hours later, cells were fixed overnight in 4% paraformaldehyde.

In RNAi experiments, cells were transfected for 72 hr with siRNA before infection. Then 3–4 hr post-infection, media containing 5 μM CldU was added. After a 1 hr incubation, the cells were fixed overnight in 4% paraformaldehyde. The next day, coverslips were washed in PBS, treated with 2 N HCl for 30 min, and washed in PBS. After a 30 min incubation in blocking buffer (3% BSA, 1% fetal bovine serum in PBS) and 10 min in blocking buffer/0.05% Triton, cells were incubated in blocking buffer containing rat anti-bromodeoxyuridine (anti-BrdU) antibodies (Abcam, ab6326, 1:400) for 1 hr. This was followed by PBS washes, secondary antibody incubation (anti-rat Alexa488, 1:400) for 30 min, PBS washes, and DAPI counterstaining. Coverslips were mounted in Mowiol.

### Statistical Analysis

All data are presented as means ± SEM and were analyzed by one-way ANOVA with Tukey’s multiple variance test using Prism 7 (GraphPad).

### Cellular Fractionation

HeLa cells (100 mm tissue culture dish per condition) were washed once in cold PBS, scraped on ice, centrifuged 4 min at 800 × *g*, and resuspended in 1 mL cold PBS. After centrifugation, the pellet was resuspended in 250 μL lysis buffer (10 mM Tris [pH 7.5], 10 mM EDTA, 150 mM NaCl, 20 μg/mL digitonin, 20 mM NaF, cOmplete Mini protease inhibitor; Roche) and incubated on a rotator for 20 min at 4°C. Nuclei and cellular debris were removed by centrifugation (3 min at 2,000 × *g*), and the resultant supernatant was cleared of any remaining cellular debris by centrifugation (10 min at 13,200 × *g*) to obtain the cytosolic fraction. The pellet was washed three times in wash buffer (10 mM Tris [pH 7.5], 10 mM EDTA, 150 mM NaCl, 20 mM NaF) before resuspension in RIPA buffer (50 mM Tris [pH 8.0], 150 mM NaCl, 1% NP-40, 0.5% Na-deoxycholate, 0.1% SDS). After incubation on rotator for 20 min at 4°C, the samples were centrifuged (10 min at 13,200 × *g*) and the supernatant was stored as a pellet fraction.

## Author Contributions

A.P. and M.W. designed the study and wrote the manuscript. A.P. performed and analyzed the experiments. A.E.R. generated the recombinant GFP-H5R virus. M.H. performed experiments leading to this study.

## Supplemental Information

Document S1. Figures S1–S3

Document S2. Article plus Supplemental Information
