# Supplementary material for: Cytoplasmic ATR Activation Promotes Vaccinia Virus Genome Replication
Source: Cell Rep. 2017 May 2;19(5):1022–32. doi: 10.1016/j.celrep.2017.04.025 (PMC5437729; doi:10.1016/j.celrep.2017.04.025)
Supplement: Document S1. Figures S1–S3 [file mmc1.pdf]

Cell Reports, Volume *19*

Supplemental Information

Cytoplasmic ATR Activation Promotes Vaccinia Virus Genome Replication

Antonio Postigo, Amy E. Ramsden, Michael Howell, and Michael Way

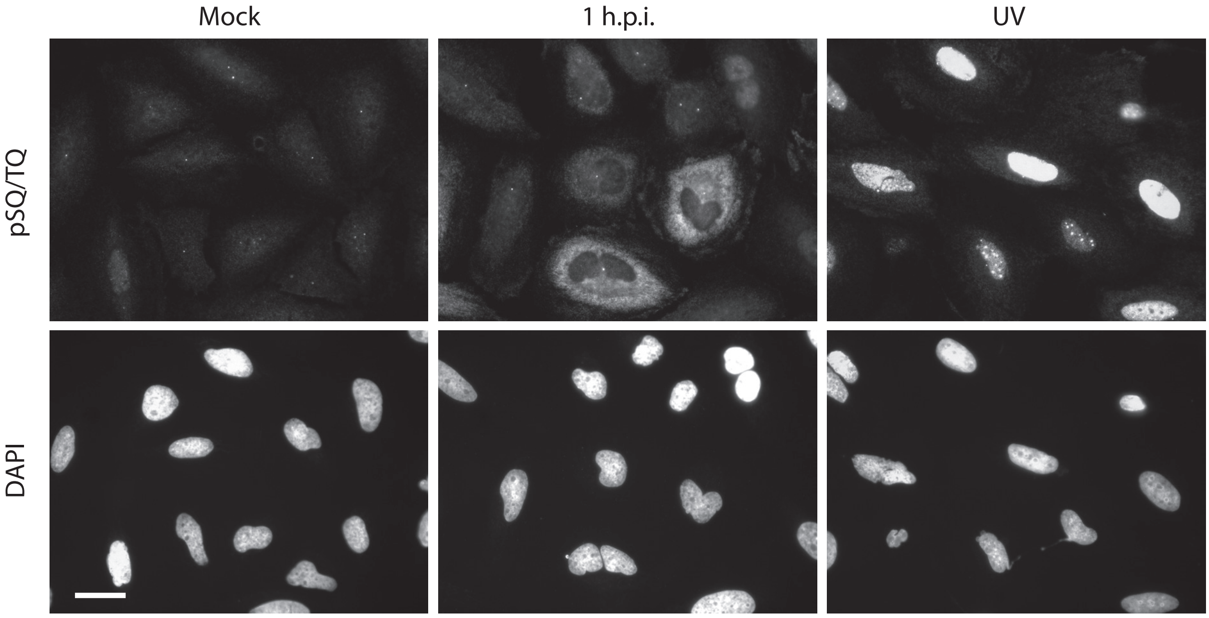

Figure S1. Vaccinia infection induces phosphorylation of SQ/TQ motifs in the cytosplasm. Related to Figure 1. Analysis of pSQ/TQ immunoreactivity reveals phosphorylation of ATR/ATM substrates in response to vaccinia infection at the indicated time post infection or UV irradiation. Scale bar = 20 µm.

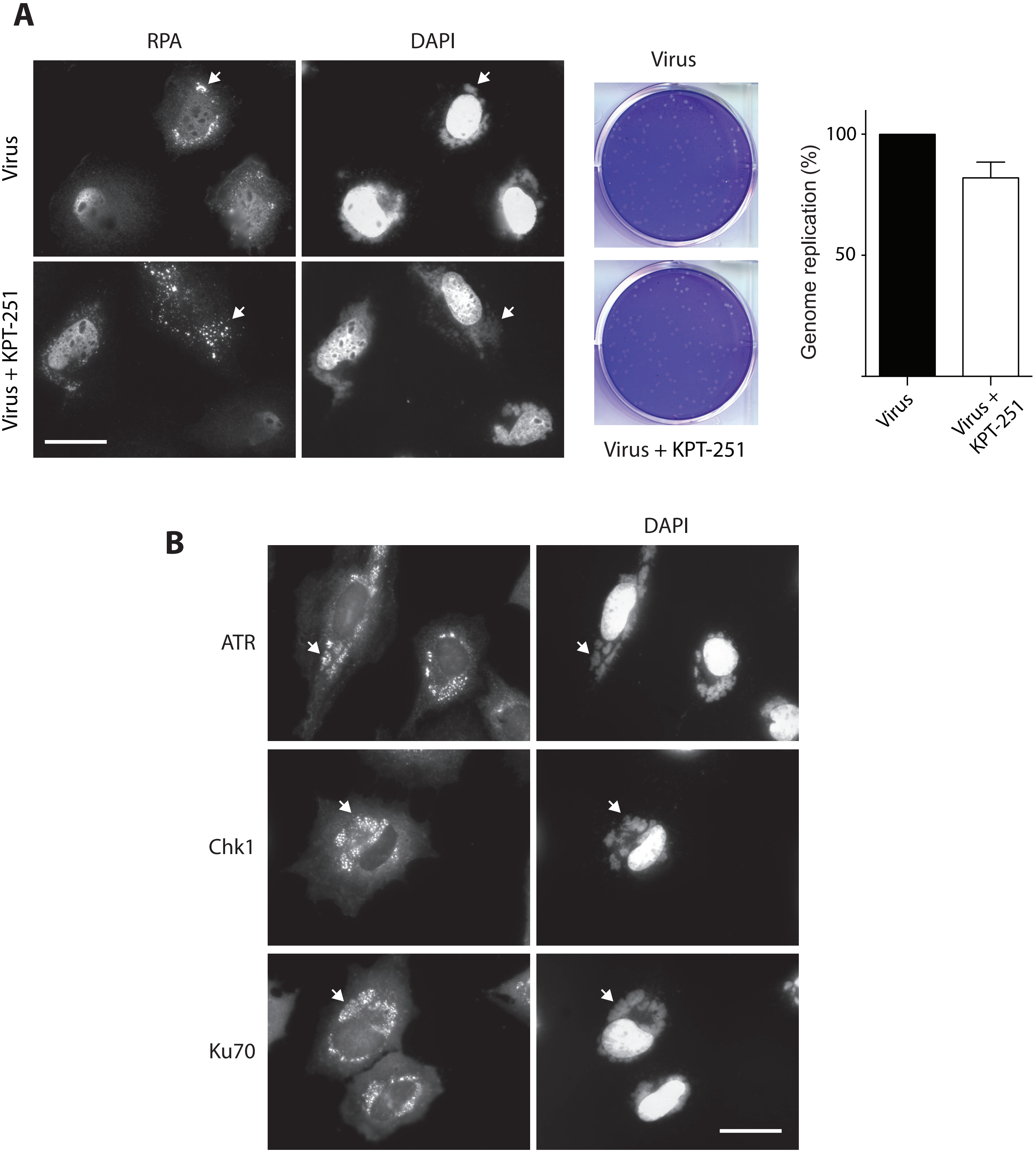

Figure S2. ATR pathway components are recruited to cytoplasmic DNA factories. Related to Figure 4.
(A) Immunofluorescence analysis reveals that inhibition of Crm-1 dependent nuclear export with KPT-251 does not block association of RPA with viral DNA factories (white arrowheads), virus growth or genome replication. **(B)** ATR, Chk1 and Ku70 associate with cytoplasmic viral DNA factories (white arrowheads). Scale bars = 20 μm.

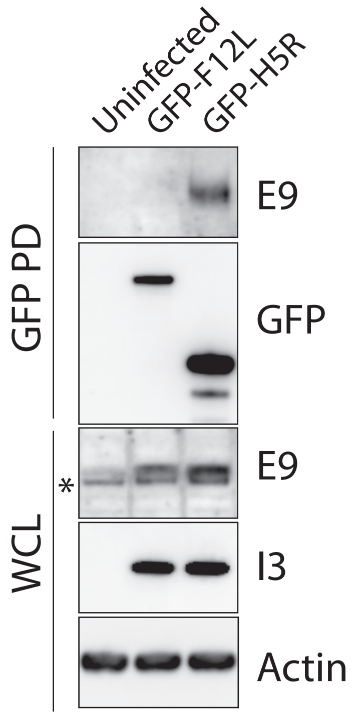

Figure S3 GFP-H5 but not GFP-F12 associates with E9. Related to Figure 5. Immunoblot analysis of GFP-TRAP pulldowns on cells infected with GFP-H5 or GFP-F12 virus for 5 hours. * indicates non-specific band migrating just below E9.
